# Brain serotonin synthesis capacity in obsessive-compulsive disorder: effects of cognitive behavioral therapy and sertraline

**DOI:** 10.1038/s41398-018-0128-4

**Published:** 2018-04-18

**Authors:** Jennifer I. Lissemore, Debbie Sookman, Paul Gravel, Alexandre Berney, Amir Barsoum, Mirko Diksic, Thomas E. Nordahl, Gilbert Pinard, Igor Sibon, Jean Cottraux, Marco Leyton, Chawki Benkelfat

**Affiliations:** 1 Department of Psychiatry, McGill University, 1033 Pine Avenue West, Montreal, QC H3A 1A1 Canada; 2 Obsessive Compulsive Disorder Clinic, Department of Psychology, McGill University Health Center, Montreal, QC H3A 1A1 Canada; 3 Department of Neurology and Neurosurgery, McGill University, Montreal, QC H3A 2B4 Canada; 4 Department of Psychiatry, Queen’s University, Kingston, ON Canada; 5 Markham Stouffville Hospital, 381 Church St., Markham, ON L3P 7P3 Canada; 6 Department of Psychiatry and Behavioral Sciences, UC Davis Medical Center, 2230 Stockton Blvd, Sacramento, CA 95817 USA; 7 Anxiety Disorder Unit, Hopital Neurologique, Lyon 1 University, 59 Boulevard Pinel, 69667 Bron, France

## Abstract

Cognitive behavioral therapy (CBT) and selective serotonin reuptake inhibitors (SSRIs) are both effective treatments for some patients with obsessive-compulsive disorder (OCD), yet little is known about the neurochemical changes related to these treatment modalities. Here, we used positron emission tomography and the α-[^11^C]methyl-l-tryptophan tracer to examine the changes in brain regional serotonin synthesis capacity in OCD patients following treatment with CBT or SSRI treatment. Sixteen medication-free OCD patients were randomly assigned to 12 weeks of either CBT or sertraline treatment. Pre-to-post treatment changes in the α-[^11^C]methyl-l-tryptophan brain trapping constant, K* (ml/g/min), were assessed as a function of symptom response, and correlations with symptom improvement were examined. Responders/partial responders to treatment did not show significant changes in relative regional tracer uptake; rather, in responders/partial responders, 12 weeks of treatment led to serotonin synthesis capacity increases that were brain-wide. Irrespective of treatment modality, baseline serotonin synthesis capacity in the raphe nuclei correlated positively with clinical improvement. These observations suggest that, for some patients, successful remediation of OCD symptoms might be associated with greater serotonergic tone.

## Introduction

Obsessive-compulsive disorder (OCD) is a chronic mental illness involving intrusive, unwanted thoughts (obsessions) and persistent mental or behavioral rituals (compulsions) that cause significant deficits in social functioning. Cognitive behavioral therapy (CBT) and selective serotonin reuptake inhibitors (SSRIs) have, in separate multicenter trials, demonstrated efficacy and tolerability in the treatment of 40–60% of OCD patients^1,2^. The success of SSRIs, relative to medications targeting neurotransmitter systems other than serotonin (5-hydroxytryptamine (5-HT)), suggests that the latter may play a role in the remediation of OCD symptoms^3,4^. Despite the documented effectiveness of these treatments, changes in neurochemistry in vivo associated with CBT or SSRI in OCD patients, including changes in the serotonergic system, remain *elusive*.

Neuroimaging and neurosurgical studies have implicated the cortico-striato-thalamo-cortical (CSTC) circuit in OCD neurobiology^5^; indeed, effective OCD treatments with either SSRIs, clomipramine, or behavior therapy, alone or in combination, have been reported to decrease abnormally elevated CSTC circuit activity^6,7^. Notably, however, conflicting findings have been reported, including increased activity within CSTC circuitry following successful OCD treatment^8^. Positron emission tomography (PET) and single photon emission computed tomography (SPECT) studies have investigated more specific aspects of neurotransmission within CSTC circuitry, including measuring 5-HT transporter (5-HTT) and receptor binding, using tracers such as [^11^C]DASB, [^123^I]β-CIT, [^11^C]McN 5652, and [^11^C]MDL100907. Pre-treatment, baseline abnormalities in 5-HTT and 5-HT_2A_ receptor availabilities within CSTC circuitry have been reported in OCD patients^9–11^, although there has been considerable variability^12–14^.

To date, few studies have investigated changes in the serotonergic system during OCD treatment. Early studies found changes in cerebrospinal fluid 5-HT metabolite levels and blood platelet 5-HTT levels pre–post treatment^15^, but these findings have not been replicated^16^, and peripheral 5-HT measures cannot be used to study brain regional changes in serotonergic functioning. To our knowledge, only one study has investigated within-subject brain regional changes in the serotonergic system in OCD patients before and after treatment: Zitterl et al. reported a significant reduction in 5-HTT availability in the thalamus/hypothalamus of OCD patients, using SPECT and [^123^I]β-CIT, following 12 weeks of clomipramine treatment^17^. Similar decreases during repeated exposure to SSRIs in various pathological and non-pathological conditions were also reviewed^17^. To our knowledge, no studies have explored the effects of CBT on the serotonergic system in OCD patients. Moreover, 5-HTT imaging has been interpreted by many to reflect density of innervation, rather than functional status per se^18^.

The PET tracer α-[^11^C]methyl-l-tryptophan (α-[^11^C]MTrp) is thought to reflect central 5-HT metabolism in humans in vivo^19,20^. α-[^11^C]MTrp is analogous to the 5-HT precursor, l-tryptophan, except that it is not incorporated into protein^21^. After crossing the blood-brain barrier, α-[^11^C]MTrp is taken up into serotonergic neurons, and ultimately is metabolized into α-M-5-HT. α-M-5-HT is not degraded by monoamine oxidase and cannot cross the blood–brain barrier, thereby accumulating in serotonergic neurons. The net blood-to-brain clearance of the tracer is used to calculate the α-[^11^C]MTrp trapping (unidirectional uptake) constant, K* (in ml/g/min). α-[^11^C]MTrp has been used to study 5-HT synthesis capacity, and more generally, 5-HT metabolism, in various patient populations^22–24^. In particular, we previously used α-[^11^C]MTrp to study baseline 5-HT synthesis capacity rates in OCD patients, and reported abnormally elevated α-[^11^C]MTrp trapping, relative to controls, in temporal, striatal, and limbic regions^25^.

As a follow-up to our baseline study of treatment-free OCD patients, the present study investigated the effects of drug treatment or CBT on brain regional 5-HT synthesis capacity. OCD patients were randomly assigned to either CBT or SSRI monotherapy (sertraline), and α-[^11^C]MTrp PET scans were repeated following 12 weeks of treatment. The goals of the present study were to (i) compare regional 5-HT synthesis capacity in OCD patients before and after treatment with CBT or sertraline, and (ii) identify brain regions where pre-treatment regional 5-HT synthesis capacity is associated with treatment outcome. Here, we expected that changes in α-[^11^C]MTrp uptake, particularly within CSTC circuitry, would relate to changes in obsessive-compulsive, but not mood, symptoms. However, as the first study of treatment-related changes in serotonin synthesis capacity in OCD patients, the current study was designed to be primarily exploratory in nature.

## Materials and methods

### Study population

Patients were referred by the OCD Clinic, Department of Psychology, McGill University Health Center (MUHC), having participated in a baseline PET study prior to beginning treatment^25^. Exclusion criteria included: (1) personal or family history of Tourette’s syndrome; (2) history of other Axis I disorders, except for depression secondary to OCD, as assessed using the Structured Clinical Interview for DSM-IV Axis I Disorders (SCID)^26^; (3) current or past substance abuse or dependence; (4) current or past use of 3,4-methylene-dioxy-methamphetamine (MDMA) or methylene-dioxy-amphetamine (MDA); and (5) history of allergy or treatment resistance to sertraline. All patients, at entry into the study, were medication-free for at least 3 weeks or more than five elimination half-lives of the drug, whichever was longer. Most patients were medication-free for considerably longer; of eight patients previously treated with antidepressants, seven were drug-free >6 months at entry into the study.

After inclusion in the study, the patients were randomly assigned (using a block randomization design with blocks of 4) to receive CBT or sertraline treatment for a period of 12 weeks. OCD symptom severity, assessed using the Yale-Brown Obsessive Compulsive Scale (Y-BOCS), and depressive symptoms, estimated with the Beck Depression Inventory (BDI), were recorded by a clinician blind to the patient’s PET data approximately every 2 weeks during treatment, beginning at baseline (week 0). Following completion of the 12-week treatment study, patients in both groups were offered further treatment as clinically indicated.

All participants provided written, informed consent. The study was carried out in accordance with the Declaration of Helsinki, and was approved by the Research Ethics Committee of the Montreal Neurological Institute (MNI) and the Institutional Review Board of McGill University.

### Treatment

Patients assigned to sertraline treatment received an initial dose of 25 mg/day. Sertraline was provided in an open fashion, as 25 mg capsules ingested once daily, in the morning with food. After 1 week of treatment, unless limited by side effects, the daily dose was increased to 50 mg/day. If, after a second week, the patient’s therapeutic response did not show evidence of symptomatic improvement, this dose was increased to 100 mg/day unless limited by side effects. A third increase in dose to 150 mg/day after another 2 weeks (week 4) and a final increase to 200 mg/day (week 6) were each made if response remained unsatisfactory (<20% decrease in Y-BOCS score). The final mean ± SD daily dose of sertraline was 133 ± 52 mg/day, and all patients were prescribed a stable dose of medication during the last 2 weeks of the study.

Patients assigned to CBT received two 90-min individual sessions per week for 12 weeks. Specialized CBT was designed and administered under the close supervision of DS, an experienced OCD expert clinician and supervisor. The specialty multidimensional CBT program was individualized for each patient and included: psycho-education; cognitive therapy to collaboratively modify symptom-related appraisals and meanings of intrusive thoughts and feared situations; strategies for dysfunctional cognitive–emotional processing, intolerance of distress, and overestimation of threat; exposure and response prevention (ERP) and behavioral experiment protocols designed to optimize adaptive learning; self-directed, between-session homework with attention to treatment adherence; and interventions for relapse prevention, resilience, and self-efficacy. Therapist-assisted ERP and behavioral experiments were administered in patients’ home as needed. Interventions specifically targeted the symptom subtype characteristics for each case^27^.

### PET and magnetic resonance imaging (MRI)

PET scans were performed before and after 12 weeks of treatment. PET and MRI procedures were carried out as per Berney et al.^25^. Briefly, in order to minimize variability between scans in plasma concentrations of amino acids, such as tryptophan, a low-protein diet followed by an overnight fast was required of participants before scanning days^28^. On PET scan days, all participants tested negative on a urine drug screen sensitive to cocaine, opiates, phencyclidine, cannabinoids, barbiturates, benzodiazepines, and amphetamines (Triage Panel for Drugs of Abuse, Biosite Diagnostics, CA, USA). Additionally, women of fertile age were scanned during their follicular phase, due to previous findings of changes in serotonergic activity in different phases of the estrous cycle in rats^29^ and the menstrual cycle in women^30^.

α-[^11^C]MTrp was produced as described elsewhere^31^. PET scanning was performed using an ECAT HR+ scanner (CTI/Siemens, Knoxville, TN; 3D mode with a resolution of 5 × 5 × 5 mm full width at half maximum (FWHM)) in the late morning/early afternoon. After a transmission scan for attenuation correction using a ^68^Ge/Ga source, α-[11C]MTrp was injected intravenously over 2 min (mean ± SD = 9.6 ± 0.8 mCi), and a 60-min dynamic image acquisition scan was performed. Thirteen venous blood samples were collected to compute the α-[^11^C]MTrp input function, as described previously^32,33^. Five plasma samples were used to measure free and total plasma tryptophan concentrations using high-performance liquid chromatography.

Each participant also underwent a T_1_-weighted MRI scan for PET-MR co-registration using a 1.5 T Philips Gyroscan scanner (Philips Medical Systems, Eindhoven, Netherlands; 3D fast-field echo scan: TR = 18 ms; TE = 10 ms; FA = 30°; 256 × 256 × 160 mm matrix; 1 mm^3^ isotropic resolution).

### Calculation of α-[^11^C]MTrp trapping (K*)

The Patlak graphic approach^34^ was used to calculate absolute K* values (ml/g/min), using dynamic PET data collected 20–60 min after tracer injection^32,33^ and peripheral metabolite values. To account for any effect of global differences in α-[^11^C]MTrp trapping on regional values, relative regional K* values were calculated by normalizing absolute regional K* values to global K* values (defined as the mean K* value for gray matter). Given that both relative and absolute K* values were previously reported to be stable over several weeks within an individual^35^, we also examined within-subject changes in absolute regional K* values. Pre- and post-treatment comparisons of regional and global K* values were carried out using both Statistical Parametric Mapping (SPM) and an MRI-based region of interest (ROI) method.

#### Voxel-based analysis using SPM

Brain-wide voxel-wise analyses comparing K* values pre- and post-treatment were carried out using SPM12 (Wellcome Functional Imaging Laboratory). K* images were spatially normalized into MNI-305 stereotaxic space, using an algorithm described elsewhere^36^, and then smoothed using a 14-mm FWHM Gaussian filter to reduce the effect of anatomical variability. The *t*-test was applied voxel by voxel. The height threshold used to interpret the *t*-test in terms of probability level was set at *p* < 0.001, uncorrected for multiple comparisons, with an extent threshold of 100 voxels, as previously^25^, then at 50 voxels for exploratory analyses. The *t*-map threshold was *T*_8_ = 4.50 for responders/partial responders and *T*_5_ = 5.89 for non-responders.

#### MRI-based ROI analysis

Pre–post treatment changes in regional K* values were also analyzed using an a priori MRI-based ROI approach. Each patient’s MRI data were corrected for field inhomogeneities and spatially normalized into MNI-305 stereotaxic space. Using an automatic segmentation method^37,38^, ROIs were defined in the left and right caudate, hippocampus, inferior temporal gyrus, cingulate, lateral and medial prefrontal cortices, nucleus accumbens, putamen, and thalamus. ROIs were smoothed using a 7 mm FWHM Gaussian filter and resampled into PET acquisition space. Time–activity curves were then derived by applying the ROIs to dynamic native PET space.

## Results

### Demographics

Sixteen patients with a diagnosis of OCD as per the SCID^26^ were included in the study. After randomization, eight patients received CBT (6M/2F), and eight sertraline (6M/2F). Data from a post-treatment PET scan were not available for one male patient treated with CBT for technical reasons, therefore a total of 15 patients was included in all PET analyses (11M/4F; mean ± SD age = 34.4 ± 9.3 years).

The demographic and clinical characteristics of the OCD patients are summarized in Table 1 for each treatment subgroup, and in Supplementary Table 1 for each clinical response subgroup. No significant differences in age, Y-BOCS score, or BDI score were found prior to treatment between treatment subgroups, or between subgroups of “responders & partial responders” vs. “non-responders”.

**Table 1:** Patient demographics

Characteristic	CBT (*n* = 8)		SSRI (*n* = 8)	
Age, y				
Mean (SD)	33.7 (9.5)		33.4 (8.5)	
Range	23–53		18–45	
Responders/partial responders	4/8		6/8	
Early-onset OCD (≤10 y), No.	5		5	
Predominant compulsion, No.				
Washing	4		4	
Checking	4		4	
Lifetime history of MDE (2° to OCD symptoms), No.	2		3	
Past substance abuse, No.	0		0	
	Pre	Post	Pre	Post
Y-BOCS score, mean (SD)	23 (4.4)	15.8 (7.5)	23.6 (5.6)	14.7 (8.2)
BDI score, mean (SD)	9.8 (4.5)	6.9 (6.0)	14.1 (11.2)	7.6 (9.5)
Plasma free tryptophan, mean (SD), nmol/L^a^	10.3 (2.6)	8.4 (1.4)	9.8 (1.7)	9.3 (2.1)
Global K*, mean (SD), mL/g/min^a^	5.1 (1.3)	6.1 (1.5)	5.8 (1.3)	6.07 (2.0)
Intravenously injected, mean (SD), mCi^a^	9.3 (1.1)	9.6 (0.7)	9.6 (0.8)	9.7 (0.4)

Responders/partial responders demonstrated a >25% decrease in Y-BOCS score

*OCD* obsessive-compulsive disorder, *CBT* cognitive behavioral therapy, *SSRI* selective serotonin re-uptake inhibitor, *MDE* major depressive episode, *Y-BOCS* Yale-Brown Obsessive Compulsive Scale, *BDI* Beck Depression Inventory, *No.* number

^a^Data not included for one patient treated with CBT

### Clinical response

**Fig. 1:**
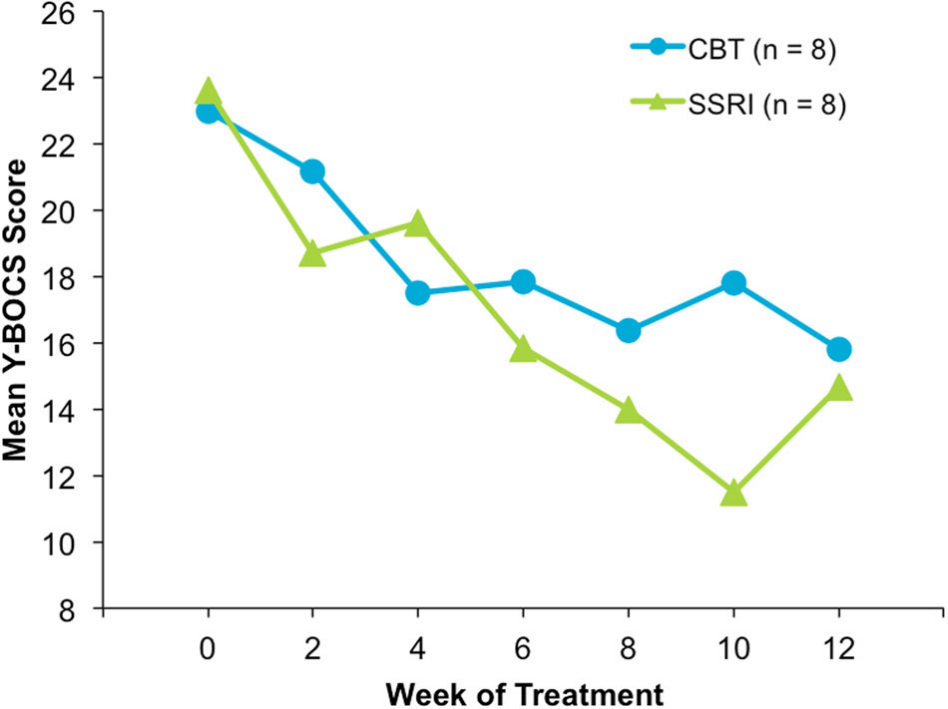
OCD symptom improvement over time with CBT or sertraline treatment. Change in mean Y-BOCS scores during 12 weeks of CBT or sertraline treatment, as measured approximately every 2 weeks. Y-BOCS Yale-Brown Obsessive Compulsive Scale, CBT cognitive behavioral therapy, SSRI selective serotonin re-uptake inhibitor

We observed a progressive improvement in mean Y-BOCS scores for both treatment groups, as illustrated in Fig. 1. At 12 weeks of continuous monitoring of clinical response, seven patients were deemed responders to treatment (≥35% decrease in Y-BOCS score), and three patients were deemed partial responders to treatment (≥25% but ≤35% reduction in Y-BOCS score)^39^; these 10 patients were combined into a group of responders/partial responders to treatment for all analyses (4 CBT/6 SSRI; mean ± SD % decrease in Y-BOCS score = 52.5 ± 20.1). Six patients were deemed non-responders to treatment (4 CBT/2 SSRI; <25% decrease in Y-BOCS score; mean ± SD % decrease = 1.9 ± 22.4). Overall, there was a significant decrease in Y-BOCS scores pre–post treatment (two-tailed paired *t*-test; *t*_15_ = 4.49, *p* < 0.001*)*; there was no significant difference between CBT and SSRI treatment groups in the pre–post % change in Y-BOCS scores (two-tailed independent *t*-test, *t*_14_ = 0.72, *p* = 0.48). In the whole sample, BDI scores pre–post treatment *decreased* significantly (Wilcoxon signed-rank test, *Z* = −2.2, *p* = 0.026), though the effect was clinically minimal.

### Global and regional α-[^11^C]MTrp trapping

Using SPM analysis, the functional images of all OCD patients from pre- and post-treatment conditions were first compared (Pre > Post and Pre < Post). No significant changes in relative (normalized) or absolute regional K* values were observed when treatment groups were combined. Accordingly, no ROIs demonstrated a significant pre–post change in relative or absolute K* values in the ROI-based analyses, and further, there was no significant pre–post change in global K* values in the whole patient sample. Similarly, when pre- and post-treatment α-[^11^C]MTrp trapping was compared within each treatment group separately (sertraline or CBT), no relative or absolute regional or global changes in K* values were identified.

**Fig. 2:**
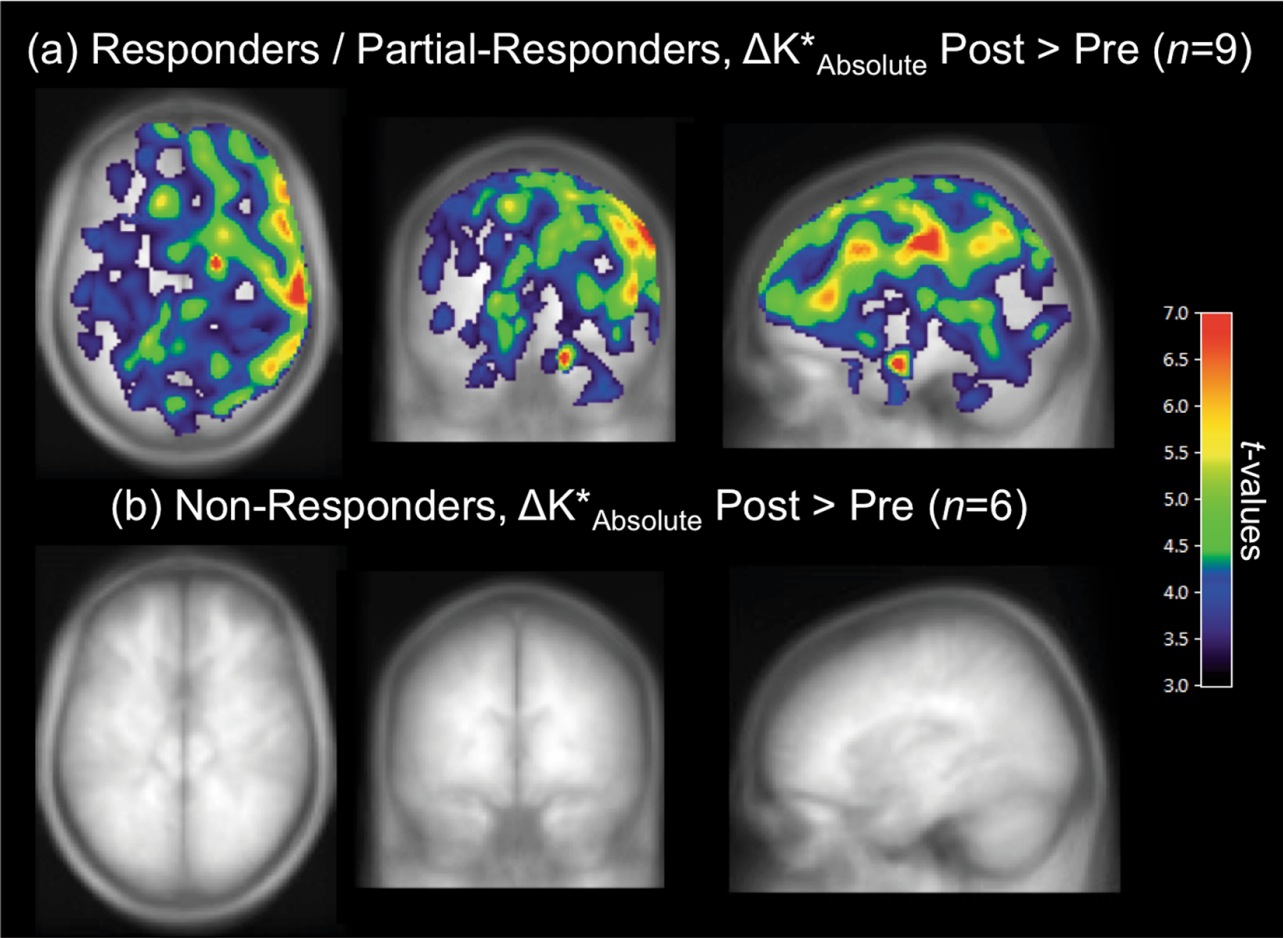
Pre–post treatment increases in serotonin synthesis capacity in responders/partial responders and non-responders. Maximum intensity projections of the *t*-values, showing brain regions where absolute K* values (K*_Absolute_) were higher post-treatment compared to pre-treatment in clinical response sub-groups. **a** Responders and partial responders to either CBT or SSRI treatment (*n* = 9) demonstrated widespread pre–post treatment increases in absolute regional K* values. **b** Non-responders (*n* = 6) did not show any significant pre–post changes in absolute regional K* values. For visualization purposes, the displayed *t*-map threshold was *T*_8_ = 3.4 for responders/partial responders and *T*_5_ = 4.0 for non-responders, with *p* = 0.005 and an extent threshold of 50 voxels

Next, we compared pre- and post-treatment α-[^11^C]MTrp trapping in the sub-sample of responders/partial responders (*n* = 9) and non-responders (*n* = 6). Using SPM and ROI-based analyses, again, no significant pre–post changes in relative regional K* values were identified in treatment responders. However, responders/partial responders demonstrated a significant increase in global K* values pre–post treatment (two-tailed paired *t*-test, *t*_8_ = 3.05, *p* = 0.016; mean increase of 29.7%, Cohen’s *d* = 1.02), whereas non-responders showed no significant treatment-related changes in global K* values (two-tailed paired *t*-test, *t*_5_ = 0.63, *p* = 0.55; mean decrease of 6.4%). Pre-treatment values of global K* did not differ significantly between responders/partial responders and non-responders. Correspondingly, voxel-wise analyses identified brain-wide increases in absolute K* values (right » left; yet, increases in absolute K* values were observed bilaterally in the ROI analyses, see Supplementary Figure 1) in responders/partial responders pre–post treatment (Fig. 2a). By contrast, no changes in absolute K* values were observed in non-responders, pre–post treatment (Fig. 2b).

A three-way Time × ROI × Response repeated measures ANOVA yielded a significant Time × Response interaction (*F*_1,13_ = 5.67, *p* = 0.033) but not a three-way interaction (*p* = 0.61), indicating that the effects did not differ in the separate ROIs. Consistent with this, the change in global K* values pre–post treatment was significantly greater in the responders/partial responders than the non-responders (two-tailed independent *t*-test, *t*_13_ = 2.37, *p* = 0.034, Hedges’ *g* = 1.25).

### Correlations between α-[^11^C]MTrp trapping and clinical scores

**Fig. 3:**
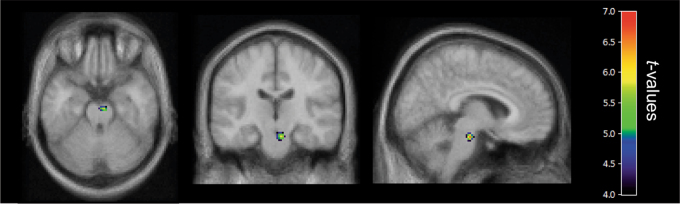
Positive correlation between baseline K* values and OCD symptom improvement. Statistical parametric maps (SPM12), with an anatomical MRI overlay, demonstrating brain regions where pre-treatment K* values correlated positively with ΔY-BOCS in the whole sample of OCD patients (*n* = 15). The *t*-map threshold was 3.85, with *p* = 0.001 and an extent threshold of 50 voxels. A significant cluster was found in the right rostral raphe nuclei (*t*_13_ = 6.66, *k* = 67 voxels, coordinates *x*, *y*, *z* = 6, −20, −22 mm)

Using SPM analysis and ΔY-BOCS scores as a covariate, we evaluated the correlation between ΔY-BOCS scores and pre-treatment K* values in the whole patient sample. Both baseline K* and ΔY-BOCS values were normally distributed. Improvement in Y-BOCS scores correlated positively with baseline α-[^11^C]MTrp trapping in the raphe nuclei within the right midbrain (*t*_13_ = 6.66, *k* = 67 voxels, coordinates *x*, *y*, *z* = 6, −20, −22 mm) independent of treatment modality (Fig. 3).

**Fig. 4:**
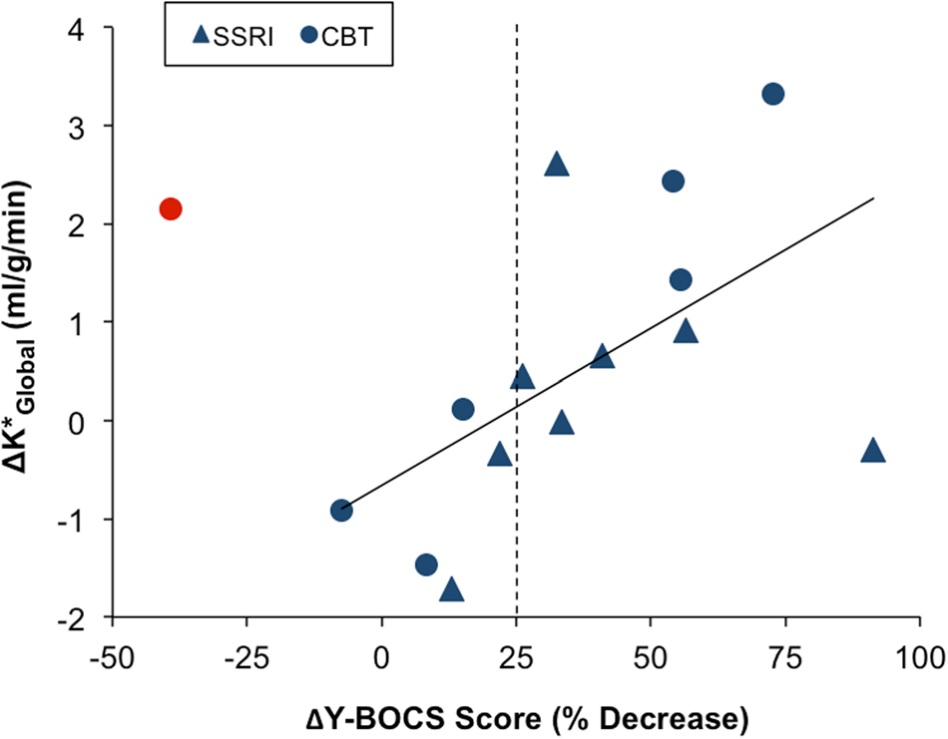
Changes in global K* values vs. changes in OCD symptom severity. Pre–post treatment changes in global K* values (ΔK*_global_) correlated positively with % decrease in Y-BOCS scores (*r*_s_ = 0.46, *p* = 0.08). Notably, there was a clear outlier in this correlation (with the outlier removed, *r*_s_ = 0.67, *p* = 0.009); the outlier (red circle) was not included in the least-squares linear fit to the data shown here. Patients treated with sertraline are represented by triangles, and patients treated with CBT are represented by circles. The ΔY-BOCS score cut-off for responder/partial responder and non-responder subgroups is indicated with the dashed vertical line. Y-BOCS Yale-Brown Obsessive-Compulsive Scale, *r*_s_ Spearman’s rank correlation coefficient

Consistent with the global K* value findings in clinical response sub-groups, pre–post treatment changes in global K* values (ΔK*_Global_) correlated positively with % decrease in Y-BOCS scores (*r*_s_ = 0.46, *p* = 0.08), as shown in Fig. 4. Notably, there was a clear outlier in this correlation, and when the outlier was removed, the correlation reached significance (*r*_s_ = 0.67, *p* = 0.009). ΔBDI scores did not correlate with pre–post treatment changes in global K* values (*r*_s_ = −0.01, *p* = 0.96) or with ΔY-BOCS scores, suggesting that concurrent changes in depressive symptoms were unlikely to have driven the reported results.

## Discussion

In this study, three distinct observations were made: (i) the SSRI sertraline and specific cognitive behavior therapy markedly reduce obsessive compulsive symptoms, (ii) this effect, though robust and significant, seldom does achieve full remission, and (iii) this effect is associated with a significant pre–post increase in whole-brain 5-HT synthesis capacity in those patients who respond to either treatment. Moreover, in the whole patient sample, increases in global 5-HT synthesis capacity correlated with reductions in OCD symptom severity. Regional changes in absolute α-[^11^C]MTrp trapping also revealed widespread increases in 5-HT synthesis capacity in responders and partial responders to either CBT or SSRI treatment (Supplementary Figure 1). Collectively, these findings support a primarily brain-wide, rather than localized, enhancement of central 5-HT synthesis capacity during effective cognitive-behavioral or sertraline (SSRI) treatment in OCD.

The reductions in obsessive-compulsive symptoms observed here are in line with previous reports of SSRI or CBT efficacy in OCD patients^1,2^. However, whereas seven patients achieved symptom remission, as defined by a Y-BOCS score ≤ 12^39^, nine patients did not remit following 12 weeks of conventional treatment. A greater understanding of the mechanisms that support symptom reduction is critical to treatment optimization, the ultimate goal being to leverage these mechanisms to achieve higher rates of remission in OCD patients. To this end, the current study emphasizes the importance of functional changes to brain 5-HT neurotransmission in the control of obsessive-compulsive symptoms, likely in conjunction with other neurotransmitters.

Independent of treatment modality, greater improvement in OCD symptoms with SSRI or CBT was also associated with higher pre-treatment 5-HT synthesis capacity in the raphe nuclei. The dorsal and median raphe nuclei are midbrain structures that contain the major serotonergic populations^40^. 5-HT is produced by the raphe nuclei, and ascending serotonergic projections from the dorsal/median raphe project to most of the brain^41^, including CSTC circuitry implicated in OCD neuropathology. The observed correlation between clinical response and baseline 5-HT neurotransmission therefore prompts speculation that elevated 5-HT synthesis capacity in the raphe nuclei prior to treatment facilitates increases in the terminal regions during clinical improvement.

Taken together with our previous findings of abnormally elevated brain regional 5-HT synthesis capacity in OCD patients at baseline^25^, the results presented here provide preliminary support for a serotonergic “braking system” operative during successful *therapeutics* in OCD. The current observations of further increases in 5-HT synthesis capacity with effective treatment support a *compensatory*, rather than pathological, role of 5-HT neurotransmission in OCD. The serotonergic braking system model posits that activation of the central serotonergic system, prior to treatment, might connote an *unsuccessful attempt* to inhibit obsessive-compulsive symptoms. CBT or SSRI exposure in OCD patients that respond to treatment could enhance this pre-existing serotonergic braking system, such that it can more effectively inhibit OC symptoms. Considering the observed association between higher pre-treatment 5-HT synthesis capacity in the raphe nuclei and greater clinical response, standard OCD treatments may provide sufficient support to this braking system in patients with higher serotonergic functioning at baseline, therefore enabling a therapeutic response. In line with the present findings, long-term treatment with SSRIs has been found to increase serotonergic neurotransmission in animals^42–44^, and, more specifically, long-term administration of sertraline has been shown to increase 5-HT synthesis in the dorsal raphe nucleus of the rat^45^. Furthermore, as reductions in brain 5-HTT expression are associated with increased serotonergic neurotransmission^46,47^, findings of reduced 5-HTT availability in OCD patients at baseline, and further reductions in 5-HTT availability with clomipramine or escitalopram treatment^17,48^, are also consistent with a serotonergic braking system.

In psychiatry, it is often assumed that abnormal brain processes will normalize to those of healthy controls with successful treatment; for example, in depression, 5-HT metabolism has been shown to be abnormally low at baseline^24^ and to normalize (increase) with antidepressant treatment^49^. Earlier brain functional imaging studies of OCD patients have also demonstrated normalization (via reduction) of glucose metabolism in OCD patients who respond to either behavioral or drug therapy^6,7^. These findings are not necessarily incongruous with the current 5-HT metabolism observations; for example, pharmacological manipulations that decrease glucose metabolism have been associated with increases in 5-HT metabolism in rodents^50^. Alternatively, the link between previous glucose metabolism findings and the current 5-HT metabolism findings in OCD could be mediated by other neural mechanisms and neurotransmitters.

Indeed, neurotransmitters seldom act in isolation. Other mechanisms and neurotransmitters, including dopamine and glutamate, have been implicated in OCD^51,52^, and likely interact with the serotonergic system in OCD^11,53,54^. Dopamine, for example, is also an important neurotransmitter in the CSTC circuit, and hyperactive dopaminergic functioning within the striatum has been associated with OCD^11^ and with compulsive behaviors in animal models of OCD^55^. Although the serotonergic findings reported here were brain-wide, our ROI analyses revealed significant increases in 5-HT synthesis capacity within CSTC circuitry following successful treatment, including in the bilateral caudate (see Supplementary Figure 1). It is possible that the 5-HT braking system counteracts dopaminergic hyperactivity within CSTC circuitry through serotonin–dopamine interactions. Specifically, increased 5-HT synthesis capacity associated with successful CBT or SSRI treatment may result in augmented 5-HT tonic inhibition of dopamine activity, and thus a reduction in compulsive symptoms. Accordingly, clinical response to SSRI therapy in OCD has been associated with reduced dopaminergic activity in the basal ganglia^56^, and can be improved using dopamine antagonist augmentation strategies^57^. Another potential mechanism underlying the observed link between elevated 5-HT metabolism and greater therapeutic response could be the known trophic properties of 5-HT in the regulation of cell proliferation, differentiation, and maturation^58^. In support of a potential neurogenic mechanism mediating the relationship between 5HT metabolism and effective treatment, 5-HT1A receptor knockout mice showed impaired neurogenesis and were insensitive to the behavioral effects of the SSRI fluoxetine^59^. It is also conceivable that non-response to SSRI or CBT may invoke mechanisms and/or neuromodulators other than serotonin. In such treatment-refractory patients, alternative therapies such as deep brain stimulation^60^ or SSRI augmentation with an antipsychotic^57^ might be beneficial.

Some limitations of the current study should be considered. (I) Although well within the range of similar studies in the field, the sample size is modest, thus replication of these findings is critical. (II) OCD research is often confounded by clinical and biological heterogeneity. Here, considerable attention was focused on preventing contamination of the biological measure of interest by non-specific factors; yet, controlling for all the non-specific factors, known (sleep, mood, motor activity, biological rhythms) or not yet known, is always difficult in clinical behavioral research, in particular with widespread neurotransmitters, such as serotonin. (III) Patients with OCD may require longer-term treatment with specialty CBT in order to optimize treatment response (see Sookman^27^ for review). Important differences in clinical and physiological indices between the treatment groups may have emerged following longer treatment duration. (IV) Several patients had been previously treated with SSRIs and/or behavioral therapy, although these patients were free of treatment for 3–90 months prior to beginning the study. Thus, we cannot formally exclude the possibility that some of the observed modifications after CBT or SSRI treatment were facilitated by previous treatments. (V) The significance of the α-[^11^C]MTrp/PET method has been discussed in some detail, and it has been suggested that the method might measure the blood–brain barrier transport of tryptophan rather than the synthesis of serotonin^61^. These reservations have been addressed in several studies and reviews from our group^19,32,33,62–65^ and others^20,66,67^, and cross-validation studies support the general consensus that brain regional α-[^11^C]MTrp trapping provides an acceptable proxy for 5-HT synthesis. (VI) It is unlikely that the observed pre–post treatment differences in regional K* values could be attributed to changes in cerebral blood flow due to treatment, since tracers with a low plasma–brain rate constant, such as α-[^11^C]MTrp, are insensitive to variations in cerebral blood flow^68^.

In conclusion, the present study did not identify region-specific changes in 5-HT synthesis capacity following treatment with either sertraline or CBT for OCD. Yet, the evidence that elevations in brain-wide serotonergic function co-varied with clinical response raises the intriguing possibility that these increases in OCD are *compensatory*. In this model, a serotonergic braking system, which is unable to sufficiently inhibit dysfunctional mechanisms prior to treatment, could become *more* engaged over the course of successful treatment with either SSRI or CBT in OCD, allowing OCD symptoms to be more effectively controlled.

## Electronic supplementary material

Supplementary Information
