# Supplementary material for: Brain serotonin synthesis capacity in obsessive-compulsive disorder: effects of cognitive behavioral therapy and sertraline
Source: Transl Psychiatry. 2018 Apr 18;8:82. doi: 10.1038/s41398-018-0128-4 (PMC5904107; doi:10.1038/s41398-018-0128-4)
Supplement: Supplementary file 1 — Supplementary Information [file 41398_2018_128_MOESM1_ESM.pdf]

Supplementary Table 1. Patient Demographics by Clinical Response Subgroup.

Characteristic	Responders / Partial-Responders (*n* = 10)		Non-Responders (*n* = 6)	
Age, y				
Mean (SD)	35.8 (9.8)		30.2 (8.6)	
Range	23-53		18-40	
Treatment group	4 CBT / 6 SSRI		4 CBT / 2 SSRI	
Early-onset OCD (≤10 y), No.	6		4	
Predominant compulsion, No.				
Washing	6		2	
Checking	4		4	
Lifetime history of MDE (2° to OCD symptoms), No.	4		1	
Past substance abuse, No.	0		0	
	**Pre**	**Post**	**Pre**	**Post**
Y-BOCS score, mean (SD)	22.5 (3.3)	10.5 (4.2)	24.7 (6.9)	23.3 (4.6)
BDI score, mean (SD)	12.5 (9.8)	6.7 (8.0)	11.0 (6.9)	8.2 (7.9)
Plasma free tryptophan, mean (SD), nmol/L [1]	10.1 (2.2)	10.0 (1.7)	10.1 (2.1)	7.6 (0.8)
Global K*, mean (SD), mL/g/min [1]	5.2 (1.6)	6.5 (1.8)	5.8 (0.4)	5.4 (1.5)
Intravenously injected, mean (SD), mCi [1]	9.5 (1.0)	9.7 (0.5)	9.7 (1.0)	9.6 (0.6)

Abbreviations: OCD, obsessive-compulsive disorder; CBT, cognitive behavioral therapy; SSRI, selective serotonin re-uptake inhibitor; MDE, major depressive episode; Y-BOCS, Yale-Brown Obsessive Compulsive Scale; BDI, Beck Depression Inventory; No., number. Responders/partial-responders demonstrated a > 25% decrease in Y-BOCS score. [1] Data not included for 1 patient treated with CBT.

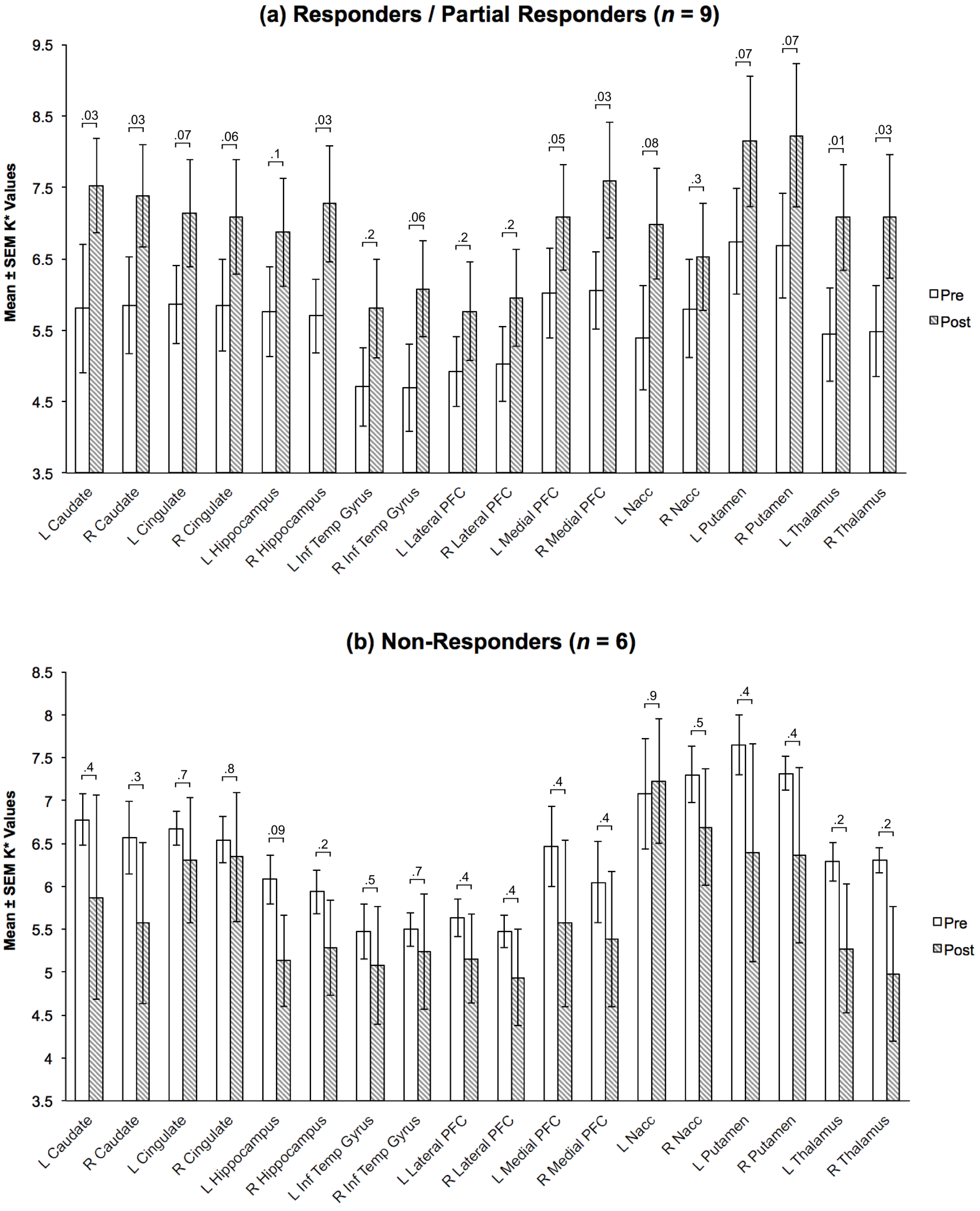

Supplementary Figure 1. Mean absolute K* values of **(a)** responders/partial responders (*n* = 9) and **(b)** non-responders (*n* = 6) at 0 weeks (Pre) and 12 weeks (Post) of either CBT or SSRI

treatment in each region of interest. The uncorrected p-values from paired t-tests comparing pre- and post-treatment K* values are indicated for each ROI. Abbreviations: L, Left; R, Right; Inf Temp, Inferior Temporal; PFC, Prefrontal Cortex; NAcc, Nucleus Accumbens.
